# Acceptability of risk stratification within population-based cancer screening from the perspective of healthcare professionals: A mixed methods systematic review and recommendations to support implementation

**DOI:** 10.1371/journal.pone.0279201

**Published:** 2023-02-24

**Authors:** Lily C. Taylor, Katie Law, Alison Hutchinson, Rebecca A. Dennison, Juliet A. Usher-Smith

**Affiliations:** 1 The Primary Care Unit, Department of Public Health and Primary Care, School of Clinical Medicine, University of Cambridge, Cambridge, United Kingdom; 2 School of Clinical Medicine, University of Cambridge, Cambridge, United Kingdom; University Medical Center of Princeton at Plainsboro, UNITED STATES

## Abstract

**Background:**

Introduction of risk stratification within population-based cancer screening programmes has the potential to optimise resource allocation by targeting screening towards members of the population who will benefit from it most. Endorsement from healthcare professionals is necessary to facilitate successful development and implementation of risk-stratified interventions. Therefore, this review aims to explore whether using risk stratification within population-based cancer screening programmes is acceptable to healthcare professionals and to identify any requirements for successful implementation.

**Methods:**

We searched four electronic databases from January 2010 to October 2021 for quantitative, qualitative, or primary mixed methods studies reporting healthcare professional and/or other stakeholder opinions on acceptability of risk-stratified population-based cancer screening. Quality of the included studies was assessed using the Mixed Methods Appraisal Tool. Data were analysed using the Joanna Briggs Institute convergent integrated approach to mixed methods analysis and mapped onto the Consolidated Framework for Implementation Research using a ‘best fit’ approach. PROSPERO record CRD42021286667.

**Results:**

A total of 12,039 papers were identified through the literature search and seven papers were included in the review, six in the context of breast cancer screening and one considering screening for ovarian cancer. Risk stratification was broadly considered acceptable, with the findings covering all five domains of the framework: intervention characteristics, outer setting, inner setting, characteristics of individuals, and process. Across these five domains, key areas that were identified as needing further consideration to support implementation were: a need for greater evidence, particularly for de-intensifying screening; resource limitations; need for staff training and clear communication; and the importance of public involvement.

**Conclusions:**

Risk stratification of population-based cancer screening programmes is largely acceptable to healthcare professionals, but support and training will be required to successfully facilitate implementation. Future research should focus on strengthening the evidence base for risk stratification, particularly in relation to reducing screening frequency among low-risk cohorts and the acceptability of this approach across different cancer types.

## Introduction

Cancer is a leading cause of global mortality with approximately 10 million cancer deaths and over 19 million new cancer diagnoses occurring in 2020 [1]. Moreover, these figures are expected to increase by almost 50% over the next two decades, amounting to a predicted 28.4 million cases in 2040 [1]. Prevention and early detection through population-based screening programmes is an effective way to reduce cancer incidence and/or mortality [2, 3]. However, as well as these benefits, cancer screening programmes are associated with costs and harms. These harms include false positive or false negative screening tests, overdiagnosis and overtreatment (where a cancer that would never cause any symptoms is diagnosed and treated), physical harms from screening or subsequent tests, and negative psychological impacts [3, 4]. Screening also incurs financial and resource costs within healthcare systems and increasing screening capacity in response to rising cancer incidence is not feasible in settings where resources are both finite and overstretched [3, 5]. Most cancer screening programmes operate a fixed regime where eligibility is based on age and/or sex and screening intervals are determined by the screening results, rather than additional individual level risk factors [5]. For example, all women aged over 25 in England are invited for cervical screening and those with a positive HPV result are invited for further screening with a reduced interval irrespective of their age or other individual level risk factors for cervical cancer. There is increasing interest in risk stratification within cancer screening programmes in order to improve the balance of benefits and harms for patients and distribute limited healthcare resources in the most efficient way [5–7].

Risk stratification involves tailoring elements of the cancer screening programme, such as test modality, screening interval or eligibility criteria, based on personal risk determined using individual level characteristics. Such an approach ensures that screening is targeted to those with the highest cancer risk whilst minimising harm to people of lower risk [5, 7]. For example, high risk individuals may be invited to attend screening from an earlier age or to attend more frequently and those of low risk may receive reduced intervention or even forgo screening entirely [6, 7].

Implementing risk stratification into cancer screening programmes could maximise diagnostic yields while using the same quantity of resources by distributing them more efficiently [5, 6]. This approach not only confers benefits to the patients undergoing screening but will also impact on healthcare professionals (HCPs) and wider stakeholders involved in screening service provision [5]. In particular, service providers and other stakeholders stand to benefit through optimising allocation of scarce resources, reduced financial burden, decreased waiting times, and potential improvements in patient compliance with screening recommendations [5]. Furthermore, there is an additional benefit of identifying suitable candidates for risk reducing interventions and enabling clinicians to use patients’ risk level in shared decision-making [5]. However, risk stratification also requires introducing complexity into screening programmes, such as increased burden relating to the engagement and risk-based management of patients, supplementary workforce training, and ethical challenges associated with endorsing reduced screening [8]. This has implications not only for those responsible for executing and monitoring the programmes, but also for those involved in delivery. HCPs within primary care and those directly involved in the programmes are also a first point of contact for individuals invited to take part in screening.

As well as being safe, affordable, and efficient, screening programmes must also be acceptable to all those involved from a clinical, social, and ethical perspective if they are to be successful [9]. Therefore, the views of HCPs on the acceptability of using risk stratification within population-based cancer screening programmes must be understood to facilitate successful development and implementation of risk-stratified interventions [7, 8, 10, 11]. Previous reviews have identified a lack of evidence surrounding acceptability and expressed a need for greater understanding of HCPs’ perspectives on acceptability, including anticipated organisational barriers and facilitators [8, 12, 13]. Furthermore, risk stratification represents a complex, large-scale change for health systems and may be influenced by organisational constraints and challenges. As such there is a need to understand acceptability within the wider organisational and structural healthcare landscape and to engage stakeholders across all areas [6, 12]. This review aims to explore whether risk stratification within population-based screening programmes is acceptable to HCPs and to identify any requirements for successful implementation.

## Methods

We performed a systematic literature review in line with a previously established study protocol (PROSPERO 2021 CRD42021286667). The methods used for the systematic literature review detailed here were the same as those used for a parallel review focused on the acceptability of risk stratification within cancer screening programmes from the perspective of the general public as detailed in the aforementioned study protocol.

### Search strategy

We performed an electronic literature search of MEDLINE, Embase, Web of Science and PsycINFO from the 1^st^ of January 2010 to the 31^st^ of November 2021 using a combination of title and abstract search terms and MeSH terms including ‘risk stratification’, ‘cancer’, ‘screening’, ‘acceptability’ and related synonyms (see S1 Table for the full search strategy). The date was restricted based on the results of preliminary searches and in order to capture contemporary views towards risk-stratified cancer screening due to the advances made in identifying and sequencing genetic variants and their use in cancer risk prediction modelling [14, 15].

### Study selection

We included English language studies that were published in peer reviewed journals that fulfilled the following eligibility criteria:

- Quantitative, qualitative, or primary mixed methods studies
- Presented in the context of population-based screening
- Specific to risk-stratified cancer screening, where risk stratification is defined as including two or more individual level risk factors beyond age and sex, including phenotypic or genetic factors, in combination to systematically determine elements of the screening programme according to individual risk
- Include healthcare professional, health service provider, and/or other stakeholder opinions on acceptability, where acceptability is defined according to the Theoretical Framework of Acceptability (TFA) as *“A multi-faceted construct that reflects the extent to which people delivering or receiving a health intervention consider it to be appropriate*, *based on anticipated or experienced cognitive and emotional responses to the interventions”* [16].

Studies conducted in the context of cancer surveillance/monitoring pathways, case finding, or investigating acceptability of non-risk-stratified screening were excluded. Studies conducted exclusively with participants who have high-risk cancer genes (e.g. *BRCA1*, *BRCA2*, *PALB2*) were excluded as these individuals are managed within surveillance programmes outside of general population-based screening.

With the support of an information specialist, one reviewer (LT) conducted the database searches, removed duplicates, and reviewed titles and abstracts for all citations. A second reviewer (RD, AH, KL) independently screened 10% of citations and any discrepancies were resolved in the presence of a third reviewer. At least two reviewers (LT, RD, AH, KL) reviewed all citations eligible for full text review. Studies that were considered ineligible by both reviewers were excluded and any areas of disagreement were resolved at consensus meetings including all researchers. The reference lists of two previous systematic reviews about risk-based breast cancer screening [17, 18] and the reference lists of all eligible studies were reviewed via the same process to identify any papers that were not found by the literature search.

### Data extraction and synthesis

A primary reviewer (LT) completed the preliminary phase of data extraction for all eligible studies using a standardised form including title, author and year, primary aim, setting, cancer type(s), sample size, demographic characteristics, study design, and method of analysis. A second stage of data extraction in which the results, themes (if applicable) and the authors’ conclusions from each included study was completed by the primary reviewer and 50% of papers underwent data extraction by a second independent reviewer (KL) to reduce bias.

Data extraction and synthesis followed the Joanna Briggs Institute convergent integrated approach to mixed methods systematic reviews [19, 20]. This approach was taken as it is recommended where the research question is able to be answered by both quantitative and qualitative data [19–21]. All data presented in the results section of the eligible papers were extracted directly into NVivo 12 software (QSR International Pty Ltd; released 2018), including relevant tables and supplementary results. As in the convergent integrated approach, quantitative data from quantitative and primary mixed methods studies were first extracted from each study and then transformed into qualitative statements. Each statement was a textual description, including relevant numerical results, produced by narrative interpretation of the data. These were presented alongside contextual anchors to preserve the integrity of the findings [20]. The qualitative statements generated by transforming the data in this way were combined with the qualitative data and all were coded according to the Consolidated Framework for Implementation Research (CFIR), drawing on a ‘best fit’ approach [22–24].

The CFIR was chosen for its extensive coverage of domains having been developed from a number of other implementation frameworks, including the Diffusion of Innovations Theory [24, 25]. It is comprised of five high level domains: Intervention characteristics, Outer setting, Inner setting, Characteristics of individuals, and Process. The intervention in this case is risk-stratified screening. The outer setting includes external policies, guidance, and pressures. The inner setting refers to organisational factors such as the culture and resources available in healthcare systems. The individuals in question are the HCPs and the process relates to the spectrum of implementation activities from planning through to execution.

The ‘best fit’ approach involves coding data against an *a priori* framework and then using thematic analysis methods to accommodate any data that does not fit within the initial framework to generate a final framework that is a better fit for the data in question. Benefits of this approach include its specificity to the review context and a reduced likelihood that data will be inappropriately ‘shoehorned’ into an existing model [23]. Furthermore, this approach has been adopted frequently for analysis in systematic reviews relating to HCPs, health organisations, and wider health policy [26–28]. We began by coding the data according to the original CFIR framework and then reviewing the contents of each construct. Construct names were amended to better suit the meaning of the data within them, framework constructs with a high degree of overlap were merged, and new construct definitions were generated for any codes that did not sit comfortably in the *a priori* framework.

Once all the data had been coded against the final best-fit framework, sub-themes were synthesised via thematic analysis within each framework construct, involving the aggregation of closely related codes. To synthesise the data, we summarised the content of each sub-theme, and sought to identify and interpret areas of similarity, ambiguity, and disagreement.

The iterative process of data synthesis was conducted via a series of meetings by the first author (LT) who has limited experience of qualitative methods and a second researcher (RD) who has extensive experience in qualitative research. Initial coding and analyses were performed independently, and researchers then came together for subsequent revisions and to refine the final framework and lower-level themes.

### Quality assessment

Quality assessment was performed by two independent reviewers (LT& RD/ KL) for all eligible papers using the Mixed Methods Appraisal Tool (MMAT) [29]. The MMAT is designed for the assessment of five different study types (qualitative research, mixed methods research, quantitative descriptive studies, randomised controlled trials, and non-randomised studies) and consists of a series of screening questions that can be answered as ‘yes’, ‘no’, or ‘can’t tell’. No studies were excluded on the basis of quality.

## Results

### Study selection

**Fig 1:**
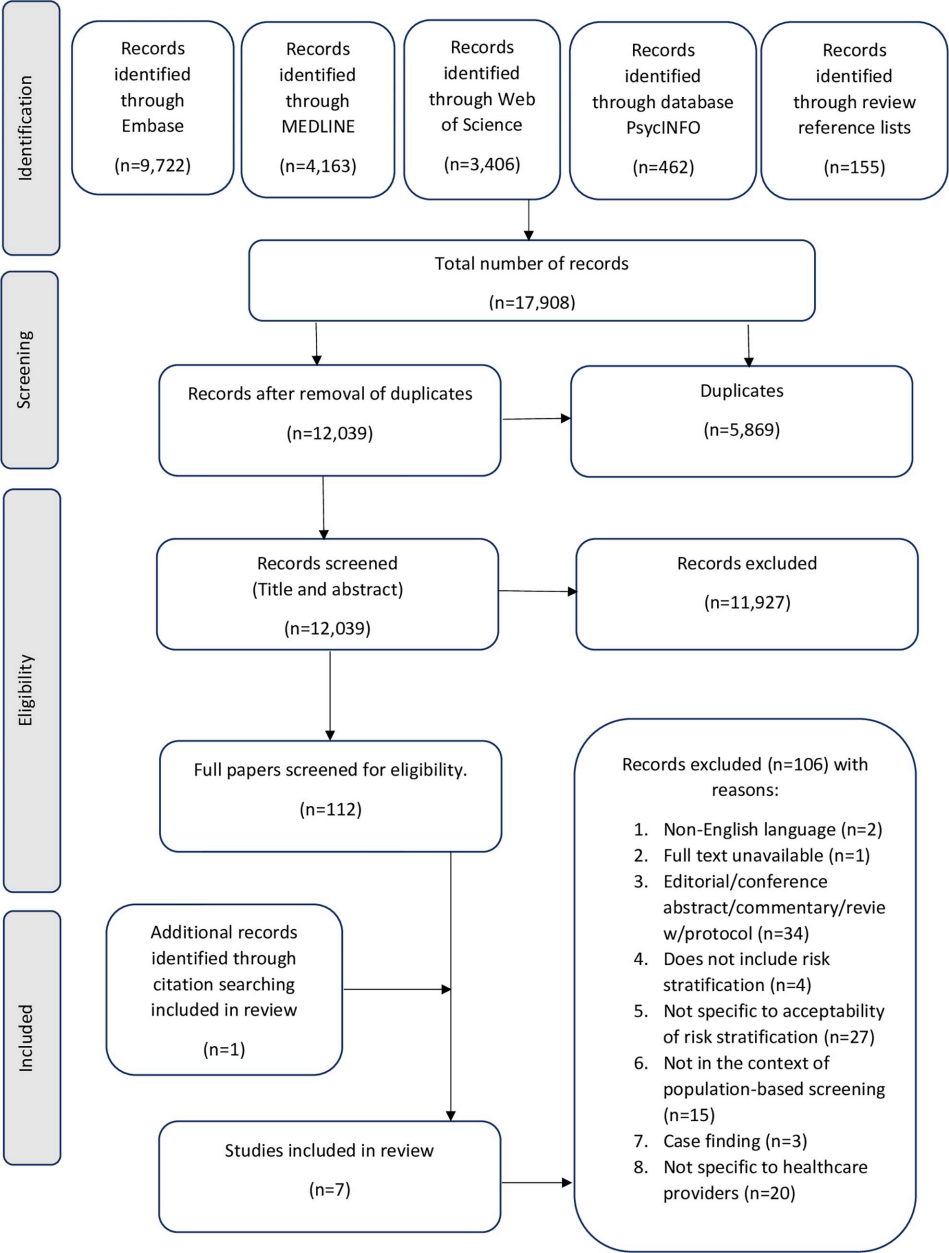
PRISMA flow diagram.

The search generated 12,039 citations, after removal of duplicates. Of these, 11,977 were excluded at title and abstract review with an agreement of 96%. A further 106 citations were excluded after full text screening and the most common reasons for exclusion at this stage were that the papers were non-empirical research or were not specific to the acceptability of risk stratification (Fig 1). An additional paper was identified through searching the reference lists of the eligible citations, resulting in a total of seven papers eligible for inclusion in the review [30–36].

### Study characteristics

The characteristics and key findings of each study are summarised in Table 1. The majority of studies were in relation to breast cancer screening (N = 6/7, 86%) [31–36], with a single study conducted in the context of ovarian cancer screening [30]. A wide variety of HCPs were involved across the eligible studies, including clinicians, researchers, and operational staff, and sample sizes ranged from 11 [33] to 829 [30] participants. Studies took place across five high-income countries: four included participants from the UK [30, 32, 34, 36], two from Canada [33, 35], and one each from Germany, Sweden, and the Netherlands, respectively [31, 32]. The majority of studies used qualitative methods in the form of interviews, focus groups, or deliberative consultation (N = 5/7, 71%) [31, 33–36]. One study utilised a quantitative cross-sectional survey design [30] and a single primary mixed methods study using digital concept mapping was also included [32].

**Table 1:** Summary of included study characteristics and key findings.

Author & year:	Setting (country):	Relevant study aim(s):	Cancer type(s):	Sample size:	Type of HCP (N):	Study design:	Method of data collection:	Themes identified: (where applicable)	Primary conclusions:
Hann (2017) [30]	UK	To investigate UK HCPs’ knowledge of ovarian cancer genetics and other risk factors, as well as self-efficacy in discussing cancer risk and genetic testing with patients, in order to identify professional training needs, and explore attitudes towards population-based genetic testing and stratified risk management.	Ovarian	829	GP (32) Genetics specialist (44) Oncologist (45) Gynaecologist (15) Nurse specialist (6) Other (4)	Quantitative	Cross-sectional survey	NA	Mixed attitudes toward risk stratification for ovarian cancer. However, most HCPs were willing to discuss management options with patients.
Fürst (2018) [31]	Germany	To assist doctors and screening participants in participatory decision-making.	Breast	15	Gynaecologist (7) GP (2) Radiologist (3 Human geneticist (1) Public health service (2)	Qualitative	Focus group	1. Assessments of individualised screening. 2. Assessments of women’s need for counselling in mammography screening 2.0. 3. Assessments of the doctors’ counselling competence. 4. Assessments of implementation of individualised screening.	Mammography screening 2.0 was viewed positively by most participants, implementation was considered more critically. Concerns expressed over time burden, competence, and guidelines.
Rainey (2018) [32]	Netherlands, UK & Sweden	To ask professionals to consider risk-based breast cancer screening and prevention from the perspective of eligible women to evaluate acceptability.	Breast	44	Netherlands (17): Researcher (7) Clinician (5) Other (5) UK (15): Researcher (3) Clinician (9) Other (5) Sweden (12): Researcher (5) Clinician (6) Other (1)	Primary mixed methods	Digital concept mapping	1. Anxiety/worry. 2. Proactive approach. 3. Reassurance. 4. Lack of knowledge. 5. Organisation of risk assessment and feedback.	Dutch, British & Swedish professionals considered women’s decision-making regarding personalised breast cancer screening similarly to women themselves. This is important for shared decision making.
Puzhko (2019) [33]	Canada	To engage health professionals in an in-depth dialog to explore the feasibility of the proposed implementation strategies for this new personalized breast cancer screening approach.	Breast	11	Genetic counsellors (3) Family physicians (8)	Qualitative	Deliberative stakeholder consultation	1. Implementation of the program: a) Introduction of the program and access to screening, b) Communicating results of individual risk estimation, c) Perspectives on women’s decision-making regarding participation in the program, d) Obstacles to using the model in a family physicians office, e) Referring women for follow-up, f) Correct interpretation of the program and its advantages, g) Uncertainty about the difference between risk assessment and screening for disease. 2. Benefits of the program: a) Benefits for HCPs, b) Benefits for women.	Risk stratification requires more clarity in communication with HCPs. Engagement of HCPs or a centralised system may be needed to ensure success of a risk stratified programme.
McWilliams (2020) [34]	UK	To elicit the views of national healthcare policy decision-makers regarding implementation of less frequent screening intervals for women at low-risk.	Breast	17	Radiologist, oncologist, radiographer, nurse, or surgeon (6) Senior academics (6) Breast screening programme operations/ management professions (5)	Qualitative	Semi-structured interviews	1. Producing the evidence defining low risk: a) Overcoming reservations about evidence accuracy, b) Determining a risk threshold and interval length, c) Risk stratification should be cost-effective. 2. The impact of risk stratification on women: a) Managing women as individuals, b) Balancing the harms and benefits, c) The ability to make autonomous decisions. 3. Practically implementing a low-risk pathway: a) Initial feasibility, b) Communication is essential, c) Considering service implications.	National healthcare policy decision makers found risk-stratified breast cancer screening generally acceptable. Before implementation there is a need to provide evidence for the accurate identification of low-risk individuals, ensure acceptability from women, demonstrate lack of harm, and ensure screening programmes are capable of facilitating multiple pathways.
Blouin-Bougie (2021) [35]	Canada	To shed light on the perceptions of healthcare professionals regarding the implementation of a BC risk stratification population-based approach.	Breast	15	GP (6) MD specialists (5) Genetic counsellors (4)	Qualitative	Semi-structured interviews	1. WHO? Target population: a) Eligible participants. 2. HOW? Clinical activities & WHAT? Associated tools: a) Identification and invitation, b) Risk assessment, c) Risk communication, d) Risk management. 3. WHICH? Conditions or prerequisites: a) Ethical approach, b) Services organisation, c) Knowledge management, d) HR administration. 4. WHY? Potential effects: a) Patients or population, b) Services delivery.	Three main conditions to facilitate acceptability of breast cancer risk stratification: respecting equity, knowledge management, and reorganising HR to optimise the workforce. Respondents welcomed risk stratification and agreed about some of the potential benefits.
Woof (2021) [36]	UK	To elicit views regarding implementing less frequent screening for low-risk women from HCPs who implement risk-stratified screening.	Breast	28	Radiographer breast imaging manager (1) Breast screening office manager (1) Breast care nurse (1) Admin and data clerk (1) GP (3) Radiographer/ mammographer (16) Cancer screening improvement lead (2) Consultant radiologist (3)	Qualitative	Focus groups & telephone interviews	1. Reservations concerning the introduction of less frequent screening: a) Low-risk screening is logical in theory, b) Questioning the reliability of risk, c) Unease towards providing screening frequency, d) Low risk is not ‘no risk’. 2. Considerations for the management of public knowledge: a) Navigating media output, b) Navigating public scrutiny, c) Impact of mixed messaging and hearsay. 3. Deliberating service implications and reconfiguration management: a) Prevalent vs incident round rollout, b) Integrating a low-risk screening interval.	Risk stratification was considered a logical step towards personalised screening. Less frequent screening was not unacceptable but was considered mindfully.

BC–breast cancer

GP–general practitioner

HCP–healthcare provider/healthcare professional

HR–human resources

Mammography screening 2.0 –individualised mammography screening

MD–Doctor of Medicine

### Quality assessment

The results of the quality assessment are presented in Table 2. The qualitative studies were found to be of high quality across all MMAT domains [31, 33–36]. The single primary mixed methods study was also of relatively high quality, scoring ‘yes’ for all MMAT domains except for ‘Do the different components of the study adhere to the quality criteria of each tradition of the methods involved?’ for which it scored ‘can’t tell’ as the risk of non-response bias was unclear [32]. Finally, the quantitative study by Hann et al., was of lower quality, scoring ‘can’t tell’ or ‘no’ in two domains as it was uncertain whether the sample was representative and there was evidence of non-response bias where self-efficacy and knowledge scores were significantly lower among those who did not complete the survey (*p* = 0.042 and *p*<0.001 respectively) [30].

**Table 2:** Results of the quality assessment using the mixed methods appraisal tool.

Author (year):	Study type:	Relevant sampling strategy?	Representative sample?	Appropriate measurements?	Nonresponse bias?	Appropriate statistical analysis?	Appropriate approach?	Adequate methods?	Findings derived from data?	Substantiated interpretation?	Coherence?	Adequate rationale?	Integrated components?	Adequate interpretation?	Inconsistencies addressed?	Quality of components?
Hann (2017) [30]	Quantitative (descriptive)	✓	?	✓	✘	✓	-	-	-	-	-	-	-	-	-	-
Furst (2018) [31]	Qualitative	-	-	-	-	-	✓	✓	✓	✓	✓	-	-	-	-	-
Puzhko (2019) [33]		-	-	-	-	-	✓	✓	✓	✓	✓	-	-	-	-	-
McWilliams (2020) [34]		-	-	-	-	-	✓	✓	✓	✓	✓	-	-	-	-	-
Blouin-Bougie (2021) [35]		-	-	-	-	-	✓	✓	✓	✓	✓	-	-	-	-	-
Woof (2021) [36]		-	-	-	-	-	✓	✓	✓	✓	✓	-	-	-	-	-
Rainey (2018) [32]	Mixed methods	-	-	-	-	-	-	-	-	-	-	✓	✓	✓	✓	?

All papers scored ‘Yes’ for screening questions 1 & 2 (S1. Are there clear research questions? S2. Do the collected data allow to address the research questions?)

✓ Yes

Χ No

? Can’t tell

### ‘Best fit’ CFIR framework

**Fig 2:**
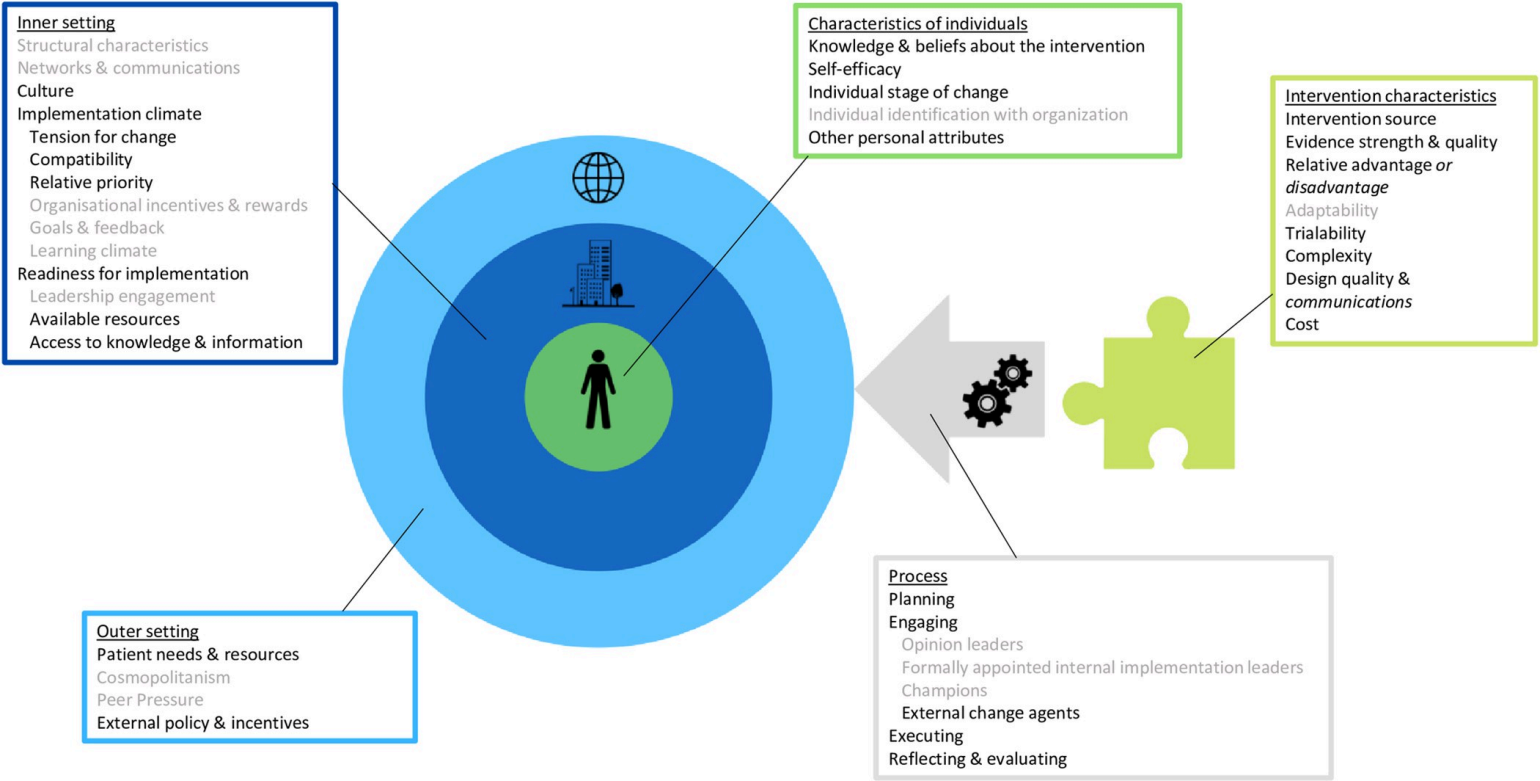
Best-fit adaptation of the consolidated framework for implementation research. Constructs are divided across 5 domains and those in grey represent themes that were not applicable to this analysis. Construct names in italics represent those that have been amended to better suit the data as part of the ‘best-fit’ approach.

The majority of themes included in the *a priori* framework were compatible with the data, however two constructs were amended to better represent their contents and several of the original CFIR constructs were not applicable to this analysis. The best fit framework we generated is presented in Fig 2. As in the original CIFR model, findings were categorised using five key domains and each of these domains contained high-level themes and lower-level sub-themes.

### Strength of the evidence

As shown in Table 3, the majority of constructs were discussed in several of the included studies, aside from the Inner setting themes ‘Tension for change’ and ‘Relative priority’ which were only briefly contributed to by two studies [31, 35]. Although these two studies were of high quality, the strength of the evidence is lacking for these particular themes. Conversely, other themes were explored across many of the included studies, most notably ‘Knowledge and beliefs about the intervention’ which was considered in detail across all seven papers, resulting in strong cumulative evidence for this construct. Three qualitative studies [33–35] considered 15 or more of the constructs, contributing greatly towards several of the themes. Furthermore, these studies were of high quality, generating robust evidence for these constructs.

**Table 3:** Strength of the evidence contributing to each construct.

		Author (year):						
		Hann (2017) [30]	Furst (2018) [31]	Rainey (2018) [32]	Puzhko (2019) [33]	McWilliams (2020) [34]	Blouin-Bougie (2021) [35]	Woof (2021) [36]
MMAT result:		Some concerns	High quality	Some concern	High quality	High quality	High quality	High quality
Intervention characteristics:	Intervention source				○	○	○	
	Evidence strength & quality			**□**	●	●	○	●
	Relative advantage or disadvantage	**□**	○		○	●	○	●
	Complexity		○	**□**	○	●	●	●
	Design quality & communications			**□**	●	○	●	●
	Cost		○	**□**		○	○	
Outer setting	Patient needs & resources			**■**	●	●	○	●
	External policy & incentives		○	**□**	○	○	●	
Inner setting	Culture		○			○	○	
	Tension for change		○					
	Compatibility					●	○	○
	Relative priority						○	
	Available resources	**□**	○		●		●	○
	Access to knowledge & information		○		○	○	○	
Characteristics of individuals	Knowledge & beliefs about the intervention	**■**	●	**■**	●	●	●	●
	Self-efficacy	**■**	○					
	Individual stage of change		○			○	○	
	Other personal attributes						○	
Process	Planning		○		○	●	○	●
	Engaging				○		○	
	External change agents					○		●
	Executing		○		○	○	○	○
	Reflecting & evaluating				○	○		

Circle = ‘yes’ for all MMAT domains

Square = ‘no’/’can’t tell’ for one or more MMAT domains

Clear = study briefly contributes to the theme

Filled = study strongly contributes to the theme

### Intervention characteristics

An overview of the synthesised findings and illustrative quotes relating to the Intervention characteristics domain is given in Table 4.

**Table 4:** Overview of the synthesised findings: Intervention characteristics.

Construct:	Sub-themes:	Illustrative quotes:
Intervention source	Views of stakeholders and the public should inform the intervention	“Participants identified individual beliefs about risk and knowledge of breast cancer and screening as key factors that will impact how women could respond to low-risk stratification. Participants felt this should guide the development of communication and information about a low-risk pathway to facilitate understanding.” [34].
	Concern about cost driving the intervention	“*… people might be concerned that the reason this was being done was to save money*, *and not necessarily for a health benefit for the wider population*, *or particularly of benefit for the women of low-risk*” (Academic) [34].
Evidence strength & quality	Concern about extending screening intervals for low-risk individuals	“*I’m not aware that it’s possible to say that because you’re a low-risk woman*, *if you do get a cancer*, *it’s going to be that kind of cancer and not this kind of cancer*” (Healthcare professional) [34].
	Stability and accuracy of variables included in risk models	“*I’m just thinking about those who might think*, *right*, *okay*, *I’ve got a low-risk*, *but what if circumstances change*? *And sometimes they might have breast cancer in the family and they might not know*, *because a lot of women don’t tell*.” (Cancer Screening Improvement Lead) [36]
	Relevance of risk models in different patients	“…genetic counsellors indicated they find RPMs generally easy to use, but not necessarily essential, because they often considered their experience and clinical judgement to be sufficient. Rather, they were concerned about the relevance of available RPMs and which of these to use for a particular patient.” [35]
Relative advantage or disadvantage	Anticipated advantages of risk stratification	“They explained that the breast screening service receives criticism for the harms it can cause and that a risk-stratified service would go some way to address this perception: “*I think we get criticised all the time for overtreatment and over diagnosis and we should be seen to be trying to personalise it a bit more*, *but we shouldn’t overthink it and overcomplicate it in the process*.” (Consultant Radiologist)” [36]
	Anticipated disadvantage of risk stratification	“*…already the UK programme gets criticised for having three yearly intervals because most European programmes have a two-year interval and they feel that 3 years*, *there’s much less of a safety net*. *You know*, *if a cancer’s missed at one screen there’s still quite a good chance that it’ll be still at an early stage at the next one two years later*. *But if the next one’s three years later there’s a bit more concern*. *So*, *I would think there’s not that much point going beyond 4 years*.” (Academic) [34].
	Anticipated neutral impact of risk stratification	“*… my sense of all of this is that what you’re doing is trying to increase the frequency for people*, *who are at higher risk and reduce it for people at lower risk […] I think probably in terms of screening visits*, *consultations and so on*, *the overall volume of work probably wouldn’t change all that much*.” (Academic) [34].
Complexity	Concerns about time and resources	“Among the major obstacles to implementation acknowledged by both types of health providers was the lack of time for PCPs during a typical 20–25-min appointment. Many felt that there is simply not enough time to introduce the program, explain risks and benefits of participation, enter the data in BOADICEA, calculate the risks, and explain the test results.” [33]
	Equity and ethical considerations	“Other participants were concerned that even if it were more feasible to introduce screening only to those entering the programme, this would create inequity of access given that all women would not have the opportunity of risk assessment.” [34]
	Lack of consensus around implementation	“There was no real consensus on how best to introduce a low-risk pathway aside from stressing the importance of obtaining the views of women themselves.” [34]
	Considerations for transitioning to risk stratification	“*… do you start the new regime for just new women coming into the programme and continue the current policy for those existing in the screening programme*? *If you do that you create an inbuilt inequality and a two-tiered service*. *Or do you allow women the choice to be given a baseline test and then a new regime*, *or allow them to continue on their old one*?” (Screening operations/management; 2027) [34].
Design quality & communications	A need for risk communication tools	“*We must have good computerized medical records and the same for everybody*, *as well as governmental tools we can access in them*. *It should be integrated in our electronic system in which there is a tab for risk assessment*. *Once you have filled it up*, *it adds to the patient’s medical records*. *It would be ideal…*” [35]
	Considerations for communicating low risk	“*I mean it’s quite a subtle message*, *isn’t it*? *For years and years we’ve been telling ladies you must go and have your screenings*, *and I think screening in the public mind is very much wrapped around screening is good always*. *I think it’s very hard to discuss subtleties of potential screening harms with people*.” (GP) [36]
Cost		“…the financial aspects for the healthcare system were also addressed: “*Because of limited resources*, *it must be considered (…) whether it is actually necessary for us to screen all women*” (public health service).” [31]

BOADICEA–Breast and Ovarian Analysis of Disease Incidence and Carrier Estimation Algorithm

FG–focus group

GP–general practitioner

PCP–primary care provider/practitioner

RPM–risk prediction model

TI–telephone interview

UK–United Kingdom

Quotes in italics represent those from HCPs. Quotes that are not in italics represent those of the author.

HCPs roles have been included where available in the original paper.

#### Intervention source

Although the source of the intervention was only considered by HCPs in three studies, those that did consider this construct emphasized the importance of involving both HCPs and the general public in all stages of development and implementation [34, 35]. Some concern was also raised about the role of cost in promoting the development of risk-stratified screening programmes and how this might be perceived by stakeholders if it were presented as one of the key drivers [34].

#### Evidence strength and quality

Two of the included studies focused specifically on introducing low-risk screening pathways. As such, much of the discussion around evidence strength was in relation to reducing screening for individuals at low risk [34, 36]. HCPs expressed doubt over the strength of the evidence for reducing breast screening of low-risk women as part of a risk-stratified pathway, particularly in relation to interval cancers and the chance of low-risk women being diagnosed with aggressive, non-hormone dependent breast cancer before they are invited for screening [34]. This was echoed by a general call for more research and modelling studies to ensure that the features of a risk-stratified programme are evidence-based [33, 36].

HCPs also highlighted the value of accurately modelling risk in developing confidence in risk estimates and in appropriately identifying risk-based cohorts [34, 36]. The variables included in risk models and the stability of risk estimates over time factored into HCPs’ consideration of the available evidence. Individuals expressed concerns that risk models may not be accurate or stable enough to allow screening to confidently be reduced for low-risk women. Although they believed the strength of the evidence was the most important factor in determining the features of a risk-stratified programme, inaccuracies in self-reported data and the unpredictable nature of the variables used in risk modelling also resulted in scepticism [36].

Additionally, there was some disparity between the priorities of different professional groups. For example, clinicians in the UK deemed concerns surrounding accuracy of risk stratification to be the most important factor in women’s decision-making process to attend screening, whereas non-clinical professionals in the UK rated this as the least important factor [32]. Genetic counsellors in particular had reservations about the applicability of risk prediction models for different patient subgroups and PCPs questioned whether the benefits conferred by risk stratification would differ according to cultural inequalities [35].

#### Relative advantage or disadvantage

Some HCPs were optimistic about the impact of risk stratification on reducing demands on staff and screening programme resources, as well as reducing heterogeneity in service provision [34, 35]. Other possible advantages of a risk-stratified approach included reducing harms such as overdiagnosis and overtreatment for low-risk individuals, particularly in the context of breast screening programmes that have received such criticism in the past [36].

HCPs further acknowledged that, in some cases, the current provision of cancer screening services is inadequate, and that introduction of a risk-stratified approach would represent an opportunity for health promotion activities [30, 31]. Genetic counsellors in particular noted that risk stratification would make risk assessment much more accessible, allowing patients to overcome lengthy waiting lists. This was thought to be particularly advantageous for those at moderate to high risk [33].

Conversely, some participants speculated that moving away from the current system to one in which screening may be reduced for some members of the population would take away the reassurance that many people gain from attending screening [36]. For established screening programmes that the public are already familiar with, changes to the screening interval might raise anxiety about cancers being missed in the interim and generate increased disapproval of the screening programme [34].

Despite these seemingly opposing opinions, some HCPs identified that the likely impact of introducing risk stratification on healthcare resources would be neutral. Although reduced screening of low-risk cohorts may have benefits for staff workload, it is probable that inclusion of higher risk cohorts would balance out this effect [34].

#### Complexity

HCPs acknowledged the complex nature of risk-stratified cancer screening compared to a standard programme and identified several barriers that would need to be addressed in order for risk stratification to be implemented in an acceptable way. These were predominantly concerns around time and resource constraints, as well as compatibility with existing screening infrastructure, which are discussed in greater detail in the ‘Compatibility’ and ‘Available resources’ sections below. The complexities of transitioning to risk-stratified screening were a fundamental concern for HCPs and a lack of consensus around how to address this was both recognised and perpetuated by participants [32, 34].

#### Design quality and communications

The communication of individual risk and the underlying motivations for risk stratification were of chief concern for many HCPs. Participants highlighted that communication styles should be tailored to individuals as much as the screening programme itself and emphasised that clarity will be essential in supporting a transition away from the current screening rhetoric [35, 36]. A need for new and adaptable risk communication tools was expressed to facilitate communication of risk, and participants felt these should be compatible with electronic systems and embedded in patient medical records [33, 35].

Rainey et al., asked HCPs to consider the importance of communication from the perspective of patients [32]. Dutch clinicians believed ‘Communicating risk’ to be the most important factor for women’s decision-making process to attend risk-stratified screening. Yet, other Dutch professionals ranked this as the least important factor for decision-making, suggesting discrepancies in how different stakeholders value and prioritise risk communication [32].

#### Cost

While some HCPs anticipated the impact of risk stratification on resources would be neutral, the potential for increased financial costs associated was remarked upon. This was particularly relevant in the context of publicly funded health services where cost has a considerable bearing on policy [34, 35]. The importance of cost-effectiveness evidence was emphasized, and participants felt this was likely to be a crucial consideration for wider stakeholders [34].

The concept of cost was also discussed in terms of time and resources. Some providers felt that risk stratification may go some way to addressing resource constraints [31]. However, others conveyed concern that risk-stratified cancer screening might actually increase costs and worsen waiting times in an already resource constrained environment [35]. Despite the importance of cost considerations for HCPs themselves, when asked to consider the views of women deciding whether to engage in risk-stratified cancer screening, Swedish healthcare providers rated financial concerns as being the least important factor to consider [32].

### Outer setting

An overview of the synthesised findings and illustrative quotes relating to the Outer setting domain is given in Table 5.

**Table 5:** Overview of the synthesised findings: Outer setting.

Construct:	Sub-themes:	Quotes:
Patient needs & resources	Anticipated psychological impact	“HCPs in The Netherlands rated anxiety/worry as most important for women’s decision-making process for participating in personalised screening (7.23/10) including statements such as: ‘Increasing the screening frequency provides insecurity’, ‘Knowing you’re high risk instils anxiety’, and ‘Having an increased risk due to non-modifiable risk factors will increase anxiety and worry’.” [32]
	Needs of low-risk individuals	“Doubts were also raised about whether low-risk women would attend subsequent screening appointments where the NHSBSP could see reduced uptake should these women feel the service is no longer applicable: “*So if you went for the initial screening for breast and you were classed as low risk*, *then you might think*, *I won’t bother again then…* “(Cancer Screening Improvement Lead)” [36]
	Public information needs & informed decision making	“*… you need to go and check with people*, *enough*, *I would say*, *to say ‘Does she understand and then can she make an informed choice*?” (Screening operations/management) [34].
External policy & incentives	A need for uniform guidelines	“*As I told you*, *there is a national committee who is working on when we should do a mammogram*, *an MRI or so on […] But it will not be complete because there is nothing about risk stratification*, *the genes or the genetics […] if you develop new tools that comprise the use of mammograms [and radiological modalities]*, *it must be congruent with the recommendations of the national committee*. *Otherwise*, *we will be confused*.” [35]

FG–focus group

HCP–healthcare professional

MRI–magnetic resonance imaging

NHSBSP–National Health Service Breast Screening Programme

PCP–primary care provider/practitioner

Quotes in italics represent those from HCPs. Quotes that are not in italics represent those of the author.

HCPs roles have been included where available in the original paper.

#### Patient needs and resources

This was a diverse construct that also contained perceived barriers and facilitators for those receiving the intervention. This construct was examined in depth by participants across five of the included studies [32–36].

When considering the psychological impact of risk stratification and receiving personalised risk estimates, HCPs tended to focus on the potential for negative ramifications. Increased anxiety, reduced reassurance, and feelings of insecurity were suggested barriers observed across all five studies [32–36]. Genetic counsellors in one study speculated that patients may experience unnecessary stress without timely and trustworthy answers to their questions about risk [33]. This was linked to their wider concerns that women who did not have a primary care provider (PCP) would have reduced access to risk stratified screening and would be less informed as a result [33]. Some participants also acknowledged the potential for positive implications such as reassurance or a sense of empowerment in knowing one’s risk level and the ability to inform other family members about risk [32, 33, 35].

Two papers focused on low-risk individuals and considered some of the potential barriers and facilitators relating to this group specifically [34, 36]. HCPs in these studies questioned the ability of low-risk individuals to understand the meaning of risk estimates and the implications for screening opportunities, as well as the potential for interval cancers and delayed diagnoses [34, 36].

Finally, HCPs explored public information needs and the role of informed decision making across all risk groups. Informed choice was generally seen as a good thing, suggesting that individuals may want to be informed of the advantages and disadvantages of risk-stratified screening and that their questions should be appropriately addressed, and support offered where applicable [32–34, 36]. PCPs in particular felt that risk stratification could reduce anxiety levels and would be useful in shared decision-making when discussing breast cancer screening with women [33]. It was also acknowledged that different population subgroups may require different levels of support in understanding this new approach to screening [33].

#### External policy and incentives

HCPs emphasised the need for a new, homogenous set of evidence-based guidelines for risk-stratified cancer screening [31, 35]. Current guidelines were perceived as inadequate and unclear, but it was noted that guidance may need to be region-specific in practice [35].

### Inner setting

An overview of the synthesised findings and illustrative quotes relating to the Inner setting domain in given in Table 6.

**Table 6:** Overview of the synthesised findings: Inner setting.

Construct:	Sub-themes:	Quotes:
Culture	Who should interpret risk?	“*I do a lot of personal risk assessment for physicians who asked for and found difficult to answer patients’ inquiry [about BC risk]*. *But*, *is this my job*? *No*, *it is not my job to estimate BC risk for women of the general population [laughs]*. *My job is to take care of high-risk women*.” [35]
Compatibility	Compatibility with existing infrastructure	“*A screening interval longer than 3 years would cause significant service disruption due to the loss of synchronization of these less frequent screening invitations with the 3-yearly rotation of the mobile screening units*.” [36]
	Communication in the context of other screening programmes	“*… it looks like when you start introducing risk-based screening*, *there’s a whole new concept*. *I think a lot of the preparatory groundwork in terms of general principles of it is already out there*.” (Academic) [34].
Relative priority		“*Ideally*, *to be rational*, *it is women with cancer that should get access to an annual MRI in priority*, *and not her sister*. *Given that we have very limited resources*, *we have to target who can get access to services*. *You know that we do not really need a second line; we need a second line to decide who deserves to receive the services*.” [35]
Available resources	Impact on resource allocation	“Almost all interviewees raised the issue of the scarcity of human resources (12 out of 14), notably in genetics (8 out of 12). Many suggested more collaboration with HPs specialized in genetics and called for more resources, particularly in rural regions, for the approach to be ethically acceptable and feasible.” [35]
	Considerations for managing patient conversations	*“…you’re probably going to raise those questions*, *so you need to make sure that there are the resources and the capacity to have those conversations with women […] so that there is an opportunity for people*, *either*, *well*, *maybe it could be a telephone contact*, *or a face to face*, *to say*, *if you want to discuss it further*, *then you can either speak to somebody on the phone*, *or we can arrange for you to come and see somebody…”* (Academic) [35].
Access to knowledge & information		“Moreover, 11 out of 15 respondents asked for a variety of knowledge exchange tools (e.g., facilitating references from one setting to another, having electronic medical records), going beyond the clinical activities discussed above.” [35]

BC–breast cancer

HP–health professional

MRI–magnetic resonance imaging

Quotes in italics represent those from HCPs. Quotes that are not in italics represent those of the author.

HCPs roles have been included where available in the original paper.

#### Culture

Discussion around culture focussed on the roles and values held by different groups of healthcare providers, especially whose role or responsibility it is to interpret and communicate risk. It was generally accepted that risk should be interpreted by HCPs and not by lay individuals, but the supposed role of individual types of professional varied due to organisational assumptions [31, 35]. Some participants, notably general practitioners (GPs), felt that all roles relating to genetic risk should be undertaken by a geneticist, believing that interpretation of risk across all cohorts lies within their remit [35]. Conversely, geneticists themselves were content to counsel high risk individuals but felt that conversations about average risk patients lay outside of their domain and belonged within primary care [35].

Rainey et al. reported other discrepancies across professional groups especially in the UK context, suggesting that individuals’ norms, values, and assumptions vary within the inner setting and indicating that the priorities of groups like genetic counsellors may not align with the priorities of other professionals, such a GPs [32].

#### Compatibility

When considering compatibility of risk-stratified cancer screening, HCPs predominantly debated the degree of fit between risk stratification and existing screening pathways and considered this in the context of risk in other medical settings. HCPs advocated for integration with existing screening infrastructure to minimise confusion and disruption [34–36]. However, as described when considering the complexity of risk-stratification compared with existing programmes, the introduction of a risk-stratified pathway was not discussed favourably, and participants had doubts about feasibility when compared to a more simplistic programme [34, 36]. Notably, one participant felt that a risk-stratified programme would in fact be less challenging than anticipated as patients are already to exposed other risk-based health principles, for example antenatal screening [34].

#### Available resources

Many HCPs considered the level of resources available for implementation and sustained use of risk-stratified cancer screening programmes. Concerns about the ability for current resources to meet the demands of risk-stratified screening were present, alongside ideas of equity and managing the capacity for patient conversations [30, 33, 35, 36]. Participants were unsure how resources could be equitably and efficiently distributed, especially in relation to the availability and distribution of human resources in the field of genetics [33, 35].

Managing patient conversations was a key consideration across all risk groups, especially for those of low risk in comparison with other cohorts [35]. Providers felt that a new risk-based system would raise patient queries, necessitating more time for consultations, and implied that the healthcare system has an obligation to provide those answers [35].

As well as organisational assumptions around HCPs’ perceived roles within a risk-stratified programme, the availability of resources also influenced thoughts on whose responsibility risk communication should be. Despite the belief that conversations around risk should take place, HCPs were unsure whose responsibility this should be, given the time constraints and considerable workload experienced by individuals working in the health system [36]. Hann et al., found that participants who reported being unwilling to discuss risk status attributed this to a lack of available resources, including time [30]. PCPs felt that a lack of time was one of the most important barriers to risk-stratified screening, voicing fears that interpreting and explaining risk results may be too time consuming to address in a typical appointment [33]. This issue was also acknowledged in discussions with genetic counsellors and was strongly re-iterated by staff within management and operational roles [33, 34].

### Characteristics of individuals

An overview of the synthesised findings and illustrative quotes relating to the Characteristics of individuals domain is given in Table 7.

**Table 7:** Overview of the synthesised findings: Characteristics of individuals.

Construct:	Sub-themes:	Quotes:
Knowledge & beliefs about the intervention	Positive beliefs about risk stratification	“The majority of HCPs agreed that risk stratification for ovarian cancer would help identify those in most need of screening (89.8%, N = 131). 63.7% (N = 93) felt it would give patients a sense of control over their health. 71.9% (N = 105) of HCPs felt patients would be reassured by being stratified into a low-risk group.” [30]
	Negative beliefs about risk stratification	“45.2% (N = 66) of HCPs felt being stratified into a low-risk group would give patients a false sense of security. 43.1% (N = 63) of HCPs felt that being stratified into a high-risk group would have a negative impact on wellbeing. 34.4% (N = 50) of HCPs felt that being stratified into an intermediate-risk group would have a negative impact on wellbeing.” [30]
Self-efficacy		“88.3% of HCPs reported that they would be probably or definitely willing to discuss stratified interventions for patients at low risk of ovarian cancer. 85.0% of HCPs reported that they would be probably or definitely willing to discuss stratified interventions for patients at intermediate risk of ovarian cancer. 82.2% of HCPs reported that they would be probably or definitely willing to discuss stratified interventions for patients at high risk of ovarian cancer.” [30]
Individual stage of change		“All respondents (15 out of 15) talked about the usefulness of a clinical questionnaire to assess BC risk and determine the need for further evaluations or genetic testing. This appears to be well integrated into their practice, but the collection of risk factors, notably family history, varies across settings, specialties, and HPs.” [35]
Other personal attributes		*“… the government does not pay for mutation on PALB2 because it is not considered as a high-risk gene*. *So*, *there are at-risk women for whom we do nothing*. *And for me*, *this is a problem because it is recognized as a BC risk and there are available interventions*. *But who decides when the risk is sufficiently high to do something*?!*”* [35]

BC–breast cancer

HCP–healthcare professional/provider

HP–health professional

Mammography screening 2.0—individualised mammography screening

PCP–primary care provider/practitioner

Quotes in italics represent those from HCPs. Quotes that are not in italics represent those of the author.

HCPs roles have been included where available in the original paper.

#### Knowledge and beliefs about the intervention

HCPs’ knowledge and beliefs about the intervention were discussed widely across all of the included studies and can broadly be categorised as either positive or negative beliefs about risk stratification of cancer screening programmes, overlapping with considerations around the relative advantage or disadvantage.

One paper in particular noted that PCPs were confused about the difference between participating in risk assessment and participating in the screening programme itself and this influenced their beliefs about the benefits and harms of a risk-stratified programme [33]. This misunderstanding continued throughout the study, and it was clear that PCPs would benefit from having terminology associated with risk stratification clearly defined [33]. One genetic counsellor highlighted the confusion between the processes of risk assessment and diagnosis and suggested PCPs may need these clarified as distinct concepts [33]. Geneticists felt that a better understanding of such concepts may help PCPs make more appropriate and efficient referrals to tertiary genetics services [33].

#### Self-efficacy

Despite some negative beliefs about the intervention, HCPs generally felt confident in their ability to implement risk-stratified cancer screening in one survey. Over 80% of participants were willing to discuss stratified interventions for patients at all risk levels and would feel confident in explaining what low, intermediate and high-risk scores mean [30]. However, GPs ranked significantly lower (*p*<0.001) than other clinicians in terms of self-efficacy in managing consultations about cancer risk, whereas genetic specialists scored the highest overall [30]. A similar trend was reported for participants’ knowledge of ovarian cancer and genetics, where GPs scored significantly lower than other clinicians (*p*<0.001), particularly in questions relating to genetics [30]. Notably, slightly fewer HCPs felt confident in explaining an intermediate risk score to patients, and willingness to discuss interventions with high-risk individuals was reduced in comparison with intermediate and low-risk groups [30].

#### Individual stage of change

Despite being in favour of risk-stratified screening in principle, many HCPs expressed a reluctance to deviate from traditional practices, especially for long established screening programmes such as mammography screening [31, 34, 35].

### Process

An overview of the synthesised findings and illustrative quotes relating to the Process domain is given in Table 8.

**Table 8:** Overview of the synthesised findings: Process.

Planning	Which risk factors to use?	“The approach needed to invite potential eligible women remained unclear to respondents (14 out of 15); however, they proposed solutions based on available recognized BC risk factors that can be easily collected to pre-select women to be invited. The most recurrent of these were family history, breast density, and age.” [35]
Engaging	The role of external change agents	“HCPs explained that media output can ‘make or break’ public opinion, especially when communication focuses on changes to NHS services.” [36]
Executing	Necessary infrastructure for implementation	“This was always viewed as a serious risk given that current infrastructure was referred to as outdated; care should be taken to develop capable IT and administrative systems flexible enough to cope during delivery.” [34]
Reflecting & evaluating		“Should a risk-stratified approach be implemented, all participants discussed the need for monitoring procedures to ensure women are invited at the right time and allocated to correct pathways.” [34]

BC- breast cancer

EMR–electronic medical records

HCP–healthcare provider/healthcare professional

IT–information technology

NHS–National Health Service

Quotes in italics represent those from HCPs. Quotes that are not in italics represent those of the author.

HCPs roles have been included where available in the original paper.

#### Planning

The concept of planning the implementation of risk-stratified screening was explored by five studies [31, 33–36]. A key concept within this was planning which risk factors should be included to provide a risk model that is acceptable to both patients and professionals and how this information should be collected [34–36]. Additionally, HCPs advocated for pilot testing and feasibility assessment before risk-stratified screening is rolled out on a larger scale [33, 34].

#### Engaging

Engaging members of the public and increasing awareness of risk-stratified cancer screening was noted by participants in two studies [33, 35]. HCPs recognised the value of exposing both healthcare staff and the general public to the concepts of risk assessment and risk stratification to foster acceptability [33, 35].

#### External change agents

Participants were wary of the media’s ability to portray risk-stratified cancer screening negatively and the potential this has to confuse or even discourage the public from participating [34, 36]. To avoid this, HCPs suggested engaging the media with risk stratification to influence perceptions of the intervention in a positive way whilst also making sure that reliable evidence is being shared by media sources to prevent miscommunication [34, 36].

#### Executing

When reflecting on how to successfully execute risk-stratified screening, HCPs focused on the infrastructure required; this was especially important as current infrastructure was seen as outmoded and incapable of handling the necessary operations for risk stratification [33–35]. Participants called for improvements to electronic medical records systems and advised that these should be integrated with risk assessment and communication tools [33, 35].

#### Reflecting and evaluating

Reflecting and evaluating the intervention was only considered briefly in two of the included studies [33, 34]. In addition to pilot studies, HCPs observed a need for feedback and evaluation to take place at all stages of the implementation process, including ongoing monitoring after the introduction of risk-stratified programmes [33, 34].

## Discussion

### Principle findings

To our knowledge, this is the first systematic review of the acceptability of risk-stratified cancer screening across all cancer types from the perspective of HCPs. A total of seven studies were included in the review, the majority of which explored the acceptability of risk stratification in relation to breast cancer screening. Our synthesis indicates that many aspects of risk-stratified cancer screening are acceptable to HCPs and other stakeholders, who viewed it as a sensical method of achieving a better balance of harms, benefits, and costs. Additionally, we highlight a number of important facilitators and barriers that must be addressed for successful implementation, as well as considerations for associated policy. In particular, there is a need for greater evidence, particularly supporting the safety of de-escalated screening for low-risk groups, consideration of resource limitations, training and communication needs, and a need for public involvement throughout.

### Comparison with other literature

The attitudes of HCPs reported in this review are broadly consistent with the attitudes of the general public towards risk stratification of population-based cancer screening. Overall, risk stratification was seen as acceptable in principle, which is congruent with numerous studies reporting that the public are largely optimistic about risk stratification [7, 10, 37–50]. We found that HCPs had some concerns about the evidence for reducing screening for those at low-risk and the psychological and physical implications of reduced or no screening. Similar concerns have also been expressed by the public, emphasising that there are more barriers to reducing screening for low-risk patients than increasing screening for those at high risk [7, 39, 40, 42, 44–46, 48, 51–53]. A further similarity between the views of HCPs and the public is the need for clear and accessible communication with patients, to enable informed choice and to avoid worsening existing inequalities [43, 54].

Although not in the context of risk stratification per-se, changes to cervical cancer screening programmes in order to implement human papillomavirus (HPV) testing and change the intervals of screening based on HPV status have also reported similar barriers to acceptability among HCPs. In those studies, many HCPs reported positive attitudes towards increasing screening intervals for women with a normal HPV co-test whereas as others reported concerns over possible harms to patients, including increasing the risk of pre-cancers and cancer diagnoses due to longer screening intervals [55, 56]. A key barrier to changing this programme is the well-established nature of cervical screening processes and guidelines and reluctance for change, which was similarly reported by HCPs in this review [57]. However, educational interventions have been successful in improving the likelihood of HCPs finding increased screening intervals acceptable in the context of HPV testing [58]. This suggests that addressing the training and educational needs of HCPs in relation to risk-stratified cancer screening could similarly improve overall acceptability of risk stratification within cancer screening.

Smit et al. have explored HCPs views towards polygenic risk testing in clinical practice outside of the context of risk-stratified cancer screening and reported similar concerns over potentially incorrect risk estimates and the evidence underlying risk scoring [59]. These findings are relevant to risk stratification as risk prediction models may incorporate genetic data in the future. Furthermore, this study reported a lack of knowledge and self-efficacy around ordering polygenic risk tests and incorporating risk scores into clinical practice [59]. As in this review, this was particularly significant among non-genetics specialists [59]. Similarly, Smit et al. report that comprehensive guidelines, education, and supportive resources are essential pre-requisites to using genetic risk scores in clinical practice [59].

### Implications for policy, practice, and research

This review suggests that robust evidence will be required in order for HCPs to accept risk-stratified cancer screening, particularly in the case of low-risk cohorts [34, 36]. For a risk model to be considered acceptable to HCPs there should be transparency regarding the included variables, and these should be practical to obtain in order to address doubts over the inclusion of potentially inaccurate data, self-reported variables, and validation of risk prediction models across different populations. Consequently, it will be important to demonstrate the strength of the evidence to clinical staff in particular if they are to recommend reduced screening with confidence.

HCPs also acknowledged that cost-effectiveness evidence is an essential consideration for policy makers, particularly in the context of publicly funded healthcare systems. However, the participants within the included studies felt policy makers should exercise caution when conveying the financial impetus for risk stratification to the general public to avoid undermining communication of the health benefits associated with risk-stratified screening.

Alongside a need for greater evidence, a further barrier to implementation expressed in the included studies is the complex nature of risk-stratified cancer screening. Specifically, concerns about time and resource constraints need to be addressed alongside the provision of evidence that risk stratification will not have a negative impact on an already resource-limited healthcare system. A risk-stratified cancer screening programme that uses more resources is unlikely to be acceptable to HCPs. Introduction of a new screening pathway could generate a need for increased patient communication and support, and health systems should have the capacity to meet those needs before implementation. PCPs in particular felt that this should not necessarily be the responsibility of frontline staff so as not to exacerbate current workforce constraints, thus an alternative system such as engaging dedicated personnel, or an external telephone helpline should be considered [33, 36].

Many HCPs expressed positive beliefs about risk stratification, however habitual screening methods are entrenched in clinical practice, and it may take time and training for HCPs to adopt skilled and sustained use of risk-stratified screening. The roles of individuals within a risk-stratified screening programme should be clearly defined and developed in conjunction with HCPs themselves to ensure acceptability. Norms, assumptions, and cultural values appear to differ between professional groups, suggesting that professional background may impact perceptions of the intervention. For example, geneticists who are already familiar with risk assessment may adapt more readily and require less training and support that HCPs who are less experienced in this area, such as GPs. Similarly, educating HCPs about a risk-stratified programme and providing clear guidance is essential in ensuring that individuals are confident in facilitating implementation.

Some HCPs, predominantly those in primary care, may have lower self-efficacy and genetic knowledge, and be confused by the difference between participating in risk assessment, participating in screening, and being diagnosed with cancer. This underscores the need to clearly define these as distinct concepts as part of HCPs’ training and is particularly necessary as they stressed that the interpretation of individual risk should be left to clinicians and not to lay persons in case of misunderstanding or incorrect interpretation [31, 33]. Although participants found discussion around risk and communication of risk estimates to be largely acceptable, clarity around handling patients at moderate risk will be needed to increase confidence in interactions with these patients. Likewise, PCPs will require additional support in order to appropriately recommend risk-stratified interventions to high-risk patients, and to support the communication and understanding of risk which until now has typically been managed by genetic specialists.

Linked with the need for training and support, in order to enable shared decision making and discussions around risk, clinicians will require risk communication tools that complement existing technological infrastructure and provide standardised information in formats that are accessible to all patients. Infrastructure, such as electronic medical records systems, must be compatible with and capable of executing risk-stratified screening if the intervention is to be perceived as acceptable by clinicians. Moreover, these processes should be well-integrated to enable simple, rapid, and routine use within clinical practice. Alongside this, revised screening guidelines should be clearly communicated to HCPs with uniform guidance that is congruent with policy advice at all levels and is evidence based.

HCPs also considered communication with the public and noted that the influence of the media on public perceptions of risk stratification has the potential to be either positive or negative. Therefore, it will be important to engage the media as external change agents to facilitate dissemination of accurate information and generate credibility around risk-stratified cancer screening. This is particularly relevant in reassuring low-risk members of the public who face a reduction in screening opportunities in comparison with the current system [36].

Many HCPs highlighted the importance of engaging patients in shared decision-making and in consulting the public throughout all stages of development and implementation. As there is potential for both positive and negative psychological impacts of risk stratification, informed choice and patient support should be prioritised to ensure that a risk-stratified approach is understood and accepted by the public. Ultimately, the findings of this review suggest that for HCPs to find risk-stratified cancer screening acceptable, it is essential to understand whether it is acceptable from the perspective of the general public.

### Strengths and limitations

A key strength of this review is the use of an established framework, the CFIR [24]. Only minor revisions were required as part of the ‘best fit’ approach, indicating that it was a suitable framework for interpreting the data [23]. Despite this, there was a clear gap in the literature around a number of CFIR constructs. These tended to focus more on issues of implementation and systems-level change, rather than the fundamentals of acceptability, indicating a need for further research specific to the changes required across healthcare systems to implement risk stratification. The use of robust and clear definitions for key terminology such as ‘risk stratification’ and ‘acceptability’ ensured the findings of this review are specific and relevant. A further strength is the adoption of a mixed methods approach which enabled the inclusion of both quantitative and qualitative studies, providing a comprehensive review of the evidence. However, our search only yielded seven eligible papers, out of over 12,000 citations identified in the search, which highlights the challenges of conducting a systematic review in implementation research where the terminology used in reporting is often broad and heterogenous. Additionally, the quality of the included literature was found to be high, particularly across qualitative studies, increasing confidence in the overall review findings.

A limitation of this review is that the studies included are predominantly concerned with breast cancer screening. The results may, therefore, not be applicable across the spectrum of different cancer types or new cancer screening programmes and this should be addressed in future research. Similarly, all the included studies were conducted in high-income countries meaning that we are unable to comment on the views of HCPs in low-income countries that may have less well-established screening programmes and this warrants further exploration. Furthermore, it was challenging to separate the views of different groups of professionals in some cases as not all of the included studies distinguished between participants’ respective roles. We chose to exclude literature relating to familial cancer syndromes or high penetrance genes, meaning the findings of this review are not applicable to these populations. However, these groups are generally recognised as high risk and are not part of population-based cancer screening programmes. Finally, we did not include any grey or unpublished literature meaning the results of our review may be subject to publication bias. We did conduct a pilot search of the grey literature and did not identify any, we therefore believe the risk of missing data is low.

## Conclusions

This review found that risk stratification of population-based cancer screening is acceptable to the majority of HCPs. Many barriers and facilitators to implementation were considered, highlighting the importance of public involvement, training, and communication, as well as a demand for more evidence around reducing screening for low-risk groups and managing resource limitations. These points must be addressed to facilitate successful implementation of risk-stratified cancer screening.

## Supporting information

S1 Table Full MEDLINE search strategy. The full search string that was used to search MEDLINE and Embase databases, and was adapted to search Web of Science and PsycINFO.
(PDF)

S1 File PRISMA checklist. (PDF)
