# Supplementary material for: Acceptability of risk stratification within population-based cancer screening from the perspective of healthcare professionals: A mixed methods systematic review and recommendations to support implementation
Source: PLoS One. 2023 Feb 24;18(2):e0279201. doi: 10.1371/journal.pone.0279201 (PMC9956883; doi:10.1371/journal.pone.0279201)
Supplement: S1 Table — The full search string that was used to search MEDLINE and Embase databases, and was adapted to search Web of Science and PsycINFO. (PDF) [file pone.0279201.s001.pdf]

MEDLINE search:

1. “risk stratifi*”.ti,ab.	36564
2. “risk test*”.ti,ab.	506
3. “risk predict*”.ti,ab.	14348
4. “risk assess*”.ti,ab.	74952
5. personali#ed.ti,ab.	54951
6. “risk based”.ti,ab.	7194
7. Risk Assessment/	290788
8. 1 or 2 or 3 or 4 or 5 or 6 or 7	422416
9. oncology.ti,ab.	105225
10. cancer*.ti,ab.	1958861
11. neoplas*.ti,ab.	276484
12. tumo#r.ti,ab.	222621
13. Neoplasms/	455891
14. 9 or 10 or 11 or 12 or 13	2491073
15. screen*.ti,ab.	837314
16. prevent*.ti,ab.	1550634
17. Mass Screening/	110620
18. 15 or 16 or 17	2333512
19. acceptab*.ti,ab.	199611
20. perspective*.ti,ab.	370012
21. opinion*.ti,ab.	114818
22. view*.ti,ab.	506389
23. attitude*.ti,ab.	165230
24. perception*.ti,ab.	281908
25. feedback.ti,ab.	151316
26. evaluat*.ti,ab.	3884042
27. Attitude/	50137
28. Feedback/	31319
29. 19 or 20 or 21 or 22 or 23 or 24 or 25 or 26 or 27 or 28	5227806
30. 8 and 14 and 18 and 29	4782
